# The Causal Relationship between Plasma Myeloperoxidase Levels and Respiratory Tract Infections: A Bidirectional Mendelian Randomization Study

**DOI:** 10.1155/2024/6626706

**Published:** 2024-03-28

**Authors:** Xiu Liu, Chuchu Zhang, Jiajia Ren, Guorong Deng, Xi Xu, Jueheng Liu, Xiaoming Gao, Ruohan Li, Jiamei Li, Gang Wang

**Affiliations:** ^1^Department of Critical Care Medicine, The Second Affiliated Hospital of Xi'an Jiaotong University, Xi'an, China; ^2^Key Laboratory of Surgical Critical Care and Life Support, Xi'an Jiaotong University, Ministry of Education, Xi'an, China

## Abstract

**Background:**

Observational researches reported the underlying correlation of plasma myeloperoxidase (MPO) concentration with respiratory tract infections (RTIs), but their causality remained unclear. Here, we examined the cause–effect relation between plasma MPO levels and RTIs.

**Materials and Methods:**

Datasets of plasma MPO levels were from the Folkersen et al. study (*n* = 21,758) and INTERVAL study (*n* = 3,301). Summarized data for upper respiratory tract infection (URTI) (2,795 cases and 483,689 controls) and lower respiratory tract infection (LRTI) in the intensive care unit (ICU) (585 cases and 430,780 controls) were from the UK Biobank database. The primary method for Mendelian randomization (MR) analysis was the inverse variance weighted approach, with MR-Egger and weighted median methods as supplements. Cochrane's *Q* test, MR-Egger intercept test, MR pleiotropy residual sum and outliers global test, funnel plots, and leave-one-out analysis were used for sensitivity analysis.

**Results:**

We found that plasma MPO levels were positively associated with URTI (odds ratio (OR) = 1.135; 95% confidence interval (CI) = 1.011–1.274; *P*=0.032) and LRTI (ICU) (OR = 1.323; 95% CI = 1.006–1.739; *P*=0.045). The consistent impact direction is shown when additional plasma MPO level genome-wide association study datasets are used (URTI: OR = 1.158; 95% CI = 1.072–1.251; *P* < 0.001; LRTI (ICU): OR = 1.216; 95% CI = 1.020–1.450; *P*=0.030). There was no evidence of a causal effect of URTI and LRTI (ICU) on plasma MPO concentration in the reverse analysis (*P* > 0.050). The sensitivity analysis revealed no violations of MR presumptions.

**Conclusions:**

Plasma MPO levels may causally affect the risks of URTI and LRTI (ICU). In contrast, the causal role of URTI and LRTI (ICU) on plasma MPO concentration was not supported in our MR analysis. Further studies are needed to identify the relationship between RTIs and plasma MPO levels.

## 1. Introduction

Myeloperoxidase (MPO), a member of the heme peroxidase-cyclooxygenase superfamily [1], demonstrates elevated expression levels in neutrophil granulocytes, while exhibiting comparatively lower expression levels in monocytes and macrophages [2–4]. It not only catalyzes hypochlorous acid (HOCl) and reactive oxygen species (ROS) to stimulate neutrophils to phagocytose bacteria and other microorganisms [5] but also actively participates in inflammation regulation, immune cell recruitment at infection sites, and influences inflammation resolution [6].

Respiratory tract infections (RTIs) are the most common infections seen in primary care and the single most significant contributor to the overall burden of disease worldwide [7]. In the most severe instances, RTIs can progress rapidly into sepsis, multiorgan failure, and even death. Therefore, it is necessary to identify potential risk factors for RTIs and thus improve global public health. The role of MPO in inflammation, particularly in RTIs, has received more attention in recent years [8]. Several studies have reported an elevation in plasma MPO levels among patients with various respiratory conditions, such as influenza, SARS-CoV-2 infection, exacerbations of chronic bronchitis with airway obstruction, frequent exacerbations of chronic bronchitis with airway obstruction, acute respiratory distress syndrome (ARDS), and sepsis when compared to individuals without these conditions [9–15]. However, whether individuals exhibiting heightened levels of plasma MPO are more susceptible to RTIs is uncertain. Consequently, it is essential to investigate the causal effects of plasma MPO levels on RTIs at the scale of the entire population.

Mendelian randomization (MR) is an epidemiological tool using genetic variations (single-nucleotide polymorphisms, SNPs) as instrumental variables (IVs) to estimate the causal relationship between exposures and outcomes at a population level [16]. Genetic variation is randomly assigned to offspring during conception, making it less susceptible to interference from inverse associations and confounding factors compared to traditional randomized controlled trials (RCTs) [17]. A recent MR study genetically determined that elevated plasma MPO levels are causally associated with increased risks of ischemic stroke, cardioembolic stroke (CES), heart failure (HF), and atrial fibrillation (AF) [18]. However, no MR research has been conducted on establishing a causal link between plasma MPO levels and RTIs.

Therefore, the objective of this current study is to conduct a two-sample bidirectional MR study using summary-level data from a genome-wide association study (GWAS), aiming to elucidate the potential causal bidirectional relationship between plasma MPO levels and RTIs, including upper respiratory tract infection (URTI) and lower respiratory tract infection (LRTI) in the intensive care unit (ICU).

## 2. Materials and Methods

### 2.1. Study Design

**Figure 1:**
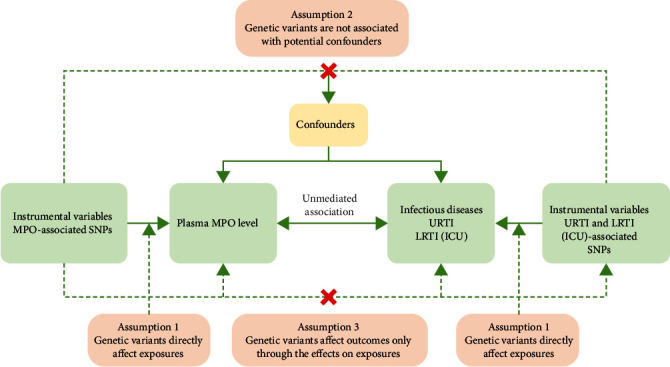
Study design for the MPO-RTIs two-sample bidirectional MR analyses. SNP, single-nucleotide polymorphism; URTI, upper respiratory tract infection; LRTI (ICU), lower respiratory tract infection in the intensive care unit.

The study design for bidirectional MR analysis is illustrated in Figure 1. Three fundamental assumptions underlie the design of MR are as follows: (1) genetic variants directly influence exposures; (2) genetic variants are not associated with potential confounders; and (3) genetic variants affect outcomes only via the effects on exposures [16]. All original studies acquired ethical approval and obtained informed consent from the participants. The data utilized in this research were readily accessible within the public domain, thus obviating the necessity for ethical approval and informed consent in accordance with the study's design. Meanwhile, the results of this study were reported in adherence to the Strengthening the Reporting of Observational Studies in Epidemiology-Mendelian Randomization (STROBE-MR) guidance from 2021 [19].

### 2.2. GWAS Data Summary for Exposures and Outcomes

After searching the IEU OpenGWAS project (https://gwas.mrcieu.ac.uk/), we identified two European-descent GWASs [20, 21] with the SNPs of plasma MPO levels and the UK Biobank database with the SNPs of URTI and LRTI (ICU). Details of these GWASs are displayed in Table 1. Folkersen et al. [20] collected 13,138,585 SNPs from 21,758 Europeans, while the INTERVAL study [21] collected 10,534,735 SNPs from 3,301 participants. Among them, the Folkersen et al. [20] study is the primary dataset, and the INTERVAL study [21] is the supplementary dataset. URTI (2,795 cases and 483,689 controls) and LRTI (ICU) (585 cases and 430,780 controls) were extracted from the UK Biobank database. All cases were diagnosed according to the International Classification of ICD-10 criteria.

### 2.3. Selection of Genetic Instruments

Based on these criteria, SNPs were used as IVs: (1) SNPs significantly correlated with plasma MPO levels (*P* < 5 × 10^−6^). This lenient threshold, due to the limited number of SNPs meeting the conventional threshold (*P* < 5 × 10^−8^), ensures the feasibility of the study [22]; (2) to eliminate linkage disequilibrium (LD), SNPs were clumped based on LD threshold (*r*^2^ < 0.001) and distance (10,000 kb); (3) we addressed SNPs not present in the outcome GWAS data by removing them, except for proxy SNPs identified with proxies = TRUE; (4) to avoid distortion of strand orientation or allele coding, we deleted palindromic SNPs (e.g., with A/T or G/C alleles); (5) SNPs without significant horizontal pleiotropy (MR pleiotropy residual sum and outliers (MR-PRESSO) global tests *P* values  > 0.05); and (6) SNPs with *F* > 10. The *F* statistics for each SNP were computed using the following formula: *F* = (*R*^2^/*k*)/([1−*R*^2^]/[*n* − *k*−1]), where *R*^2^ is the proportion of risk factor variability explained by genotype, *k* is the number of instruments used in the model, and *n* is the sample size.

### 2.4. MR Analysis

Three alternative approaches—MR-Egger regression, weighted median, and inverse variance weighted (IVW) with the random effect model—were used to investigate the genetic relationship between plasma MPO levels and the risk of respiratory infection. Since the IVW method with a random effects model assumes the validity of all SNPs utilized in the study, it can yield the most accurate estimate. Therefore, IVW with a random effects model was deemed as the principal analysis approach in this study. The remaining two methods were employed as supplementary techniques for MR analysis.

### 2.5. Sensitivity Analyses

We performed sensitivity analyses to investigate potential pleiotropic bias by several approaches: Cochrane's *Q* test, MR-Egger intercept test, MR-PRESSO global test, funnel plots, and leave-one-out analysis. Cochrane's *Q* test was used to examine the heterogeneity of the associations (a *P* value of less than 0.05 revealed heterogeneity) [23]. Horizontal pleiotropy was examined utilizing the MR-Egger intercept test, the MR-PRESSO global test (a *P* value of less than 0.05 indicated horizontal pleiotropy), and the funnel plot [24, 25]. Leave-one-out analysis to evaluate whether a single SNP drove or biased the MR estimate [16].

### 2.6. Statistical Analysis

The odds ratio (OR), beta, and 95% confidence interval (CI) were utilized to present the causal estimates, and a *P* value of less than 0.05 was deemed statistically significant. The statistical significance threshold of sensitivity analyses was set at *P* > 0.05. The forest plots, scatter plot, leave-one-out plot, funnel plot, and all statistical analyses performed in this study were performed in R (version 4.2.3) using the “TwoSampleMR” package (version 0.4.20; https://github.com/MRCIEU/TwoSampleMR).

## 3. Results

### 3.1. Genetic Prediction of Plasma MPO Levels for RTIs Risk

#### 3.1.1. IV Selection

We first selected the IVs for plasma MPO levels to assess the causal impact of plasma MPO levels on the risk of URTI and LRTI (ICU). Initially, 33 SNPs closely linked to plasma MPO levels were retrieved from the primary dataset [20] and 13 from the supplementary dataset [21]. Following strict implementation of the IV selection procedure (as described in Section 2), 28 SNPs were used as IVs between plasma MPO levels and URTI, and 29 SNPs were used as IVs between plasma MPO levels and LRTI (ICU) in the primary MR study (Supplementary Tables S1 and S3). In the supplementary MR study, 11 SNPs were identified as IVs between plasma MPO levels and URTI, as well as between plasma MPO levels and LRTI (ICU) (Supplementary Tables S2 and S4). The F statistic values were all ≥10 (Supplementary Tables S1–S4), suggesting that the possibility of weak instrument bias is slight. Supplementary Tables S8 and S9 provide the SNP filtering process.

### 3.2. Plasma MPO Levels and URTI

**Figure 2:**
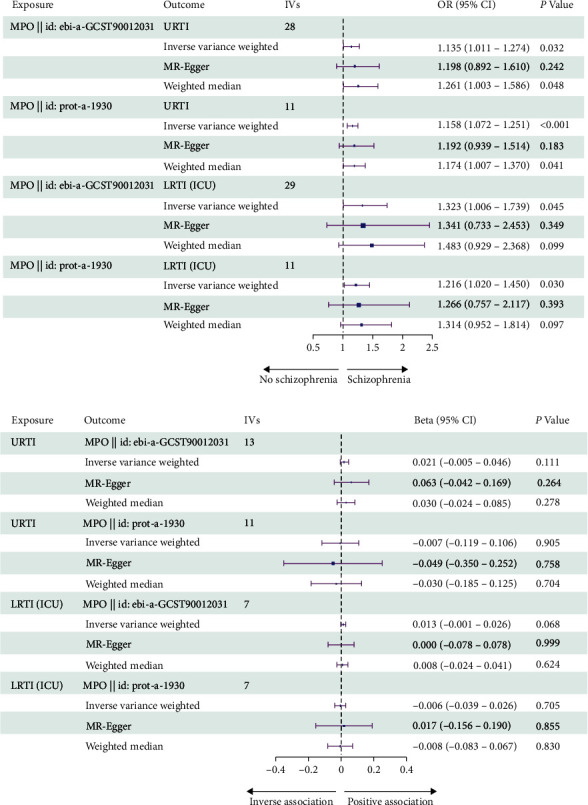
MR analysis of the effect of plasma MPO levels on RTIs (a) and MR analysis of the effect of RTIs on plasma MPO levels (b). IVs, instrumental variables; OR, odds ratio; OR (95% CI), 95% confidence interval of odds ratio. Beta (95% CI), 95% confidence interval of beta.

**Figure 3:**
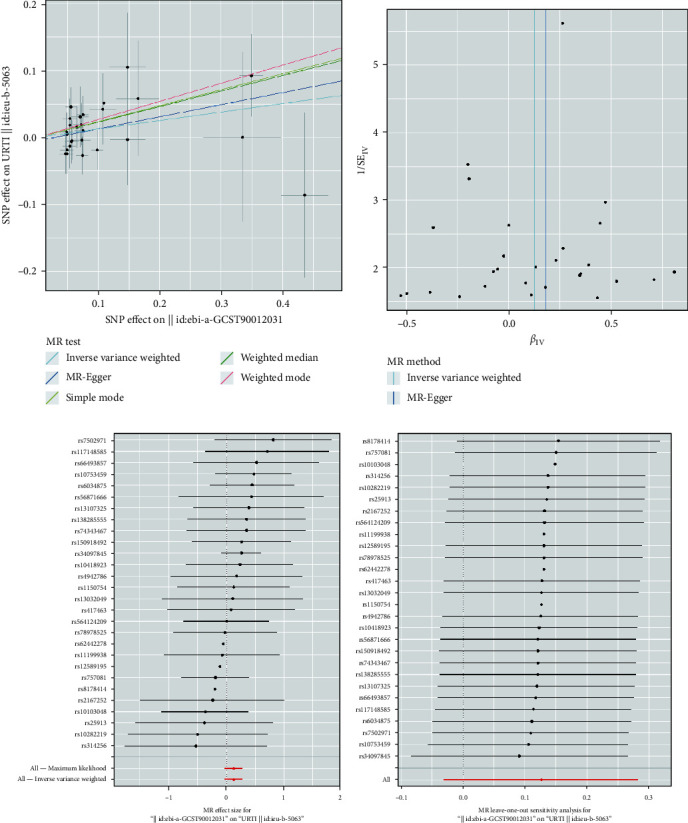
Primary MR analysis results of the causal effect of plasma MPO levels on URTI: (a) comparison of the five MR analysis methods employed; (b) funnel plot assessing directional horizontal pleiotropy; (c) forest plot displaying the effect estimates; (d) leave-one-out analyses detecting outliers.

**Figure 4:**
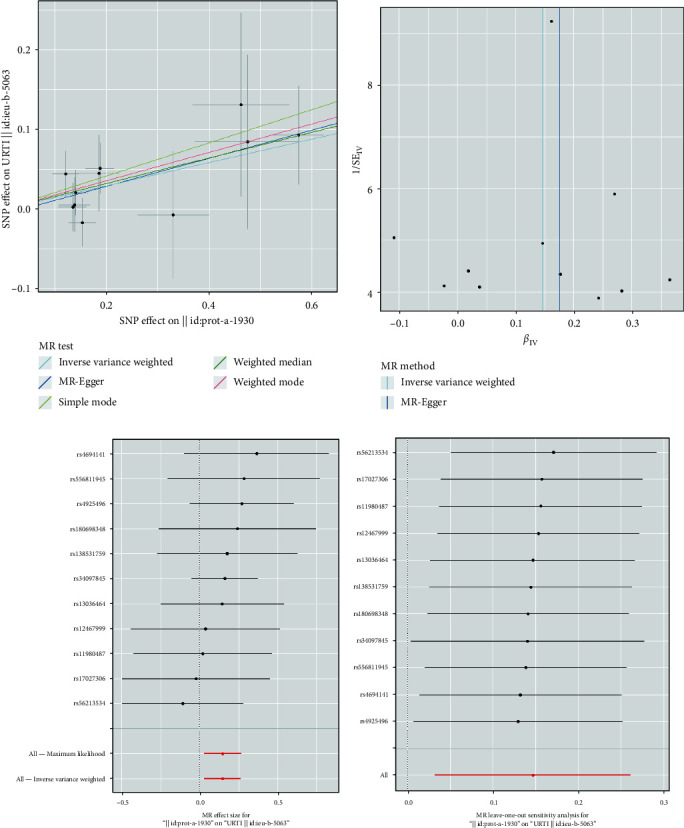
Supplementary MR analysis results of the causal effect of plasma MPO levels on URTI: (a) comparison of the five MR analysis methods employed; (b) funnel plot assessing directional horizontal pleiotropy; (c) forest plot displaying the effect estimates; (d) leave-one-out analyses detecting outliers.

In the primary MR study, based on the IVW method with the random effect model, a significant association was observed between plasma MPO levels and the risk of URTI (OR = 1.135, 95% CI = 1.011–1.274, *P*=0.032). The weighted median results were consistent with IVW (OR = 1.261, 95% CI = 1.003–1.586, *P*=0.048). While the MR-Egger analysis did not show a statistically significant relationship between plasma MPO levels and the risk of URTI (OR = 1.198, 95% CI = 0.892–1.610, *P*=0.242), the direction of effect aligned with the main analysis, especially IVW (Figure 2(a)). The supplementary MR study had a similar causal effect of plasma MPO levels on URTI. The combined OR estimated through the IVW method with the random effect model was 1.158 (95% CI: 1.072–1.251, *P* < 0.001) (Figure 2(a)). The scatter plot and forest plot showed the overall causal effect estimation and the causal effect estimation of individual SNPs (Figures 3(a) and 3(c), Figures 4(a) and 4(c)).

We performed several sensitivity analyses to evaluate the robustness of the causal effect estimates of plasma MPO levels on URTI, including horizontal pleiotropy tests, heterogeneity tests, leave-one-out analysis, and funnel plot analysis. The MR-Egger intercept tests and MR-PRESSO suggested no horizontal pleiotropy (all *P* > 0.05). Similarly, Cochran's *Q* statistics and the random-effects IVW method indicated no heterogeneity (all *P* > 0.05) (Table 2). The leave-one-out sensitivity analysis, as shown in Figures 3(d) and 4(d), demonstrated that any SNPs had little effect on the overall effect of causal relationships. Last, the funnel plots for MR analysis in Figures 3(b) and 4(b) revealed that the data points were equally distributed around the funnel, indicating that no substantial asymmetry existed and that there was no evidence of horizontal pleiotropy.

### 3.3. Plasma MPO Levels and LRTI (ICU)

**Figure 5:**
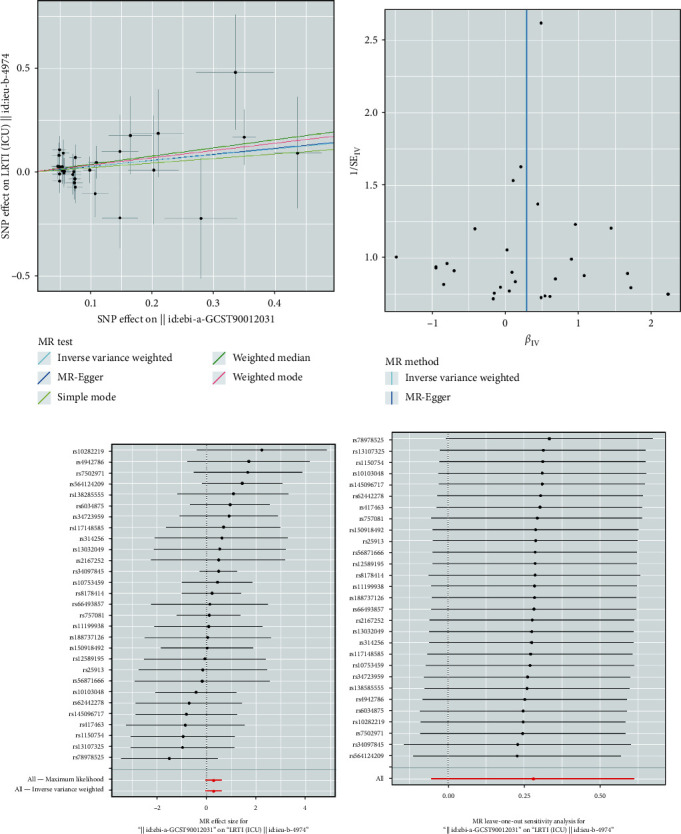
Primary MR analysis results of the causal effect of plasma MPO levels on LRTI (ICU): (a) comparison of the five MR analysis methods employed; (b) funnel plot assessing directional horizontal pleiotropy; (c) forest plot displaying the effect estimates; (d) leave-one-out analyses detecting outliers.

**Figure 6:**
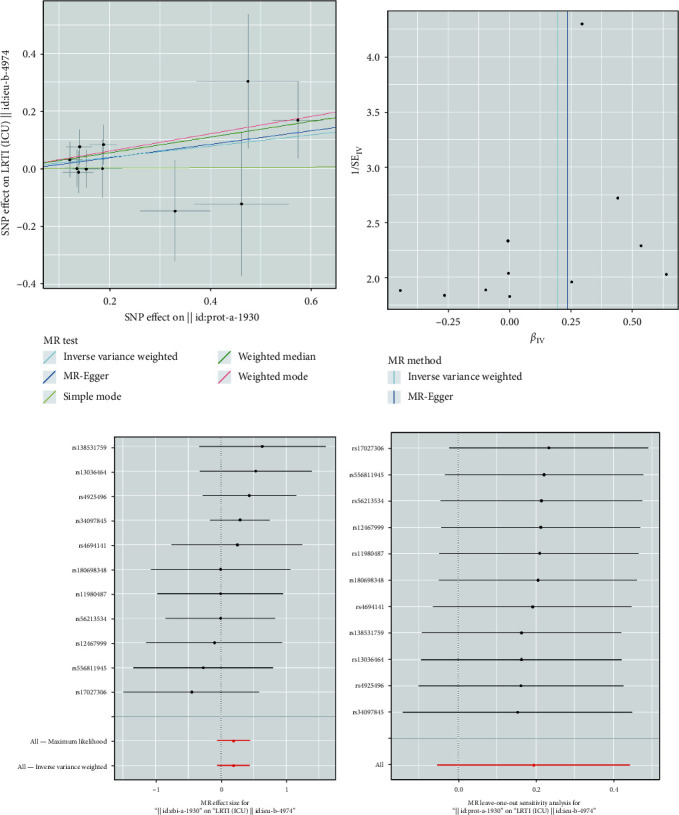
Supplementary MR analysis results of the causal effect of plasma MPO levels on LRTI (ICU): (a) comparison of the five MR analysis methods employed; (b) funnel plot assessing directional horizontal pleiotropy; (c) forest plot displaying the effect estimates; (d) leave-one-out analyses detecting outliers.

In the primary MR study, the IVW method with the random effect model suggested a significant association between plasma MPO levels and the risk of LRTI (ICU) (OR = 1.323, 95% CI = 1.006–1.739, *P*=0.045). While the weighted median (OR = 1.341, 95% CI = 0.733–2.453, *P*=0.349) and the MR-Egger analysis (OR = 1.483, 95% CI = 0.929–2.368, *P*=0.099) did not demonstrate a statistically significant relationship between plasma MPO levels and the risk of LRTI (ICU), the direction of effect was consistent with the IVW method with the random effect model (Figure 2(a)). Analogous causal effects of plasma MPO concentration on LRTI (ICU) were observed in the supplementary MR study. The combined OR estimated through the IVW method with the random effect model was 1.216 (95% CI: 1.020–1.450, *P*=0.030) (Figure 2(a)). The MR regression slopes and individual causal estimates of each SNP are illustrated in Figures 5(a) and 5(c), Figures 6(a) and 6(c).

Additionally, no evidence of heterogeneity or horizontal pleiotropy was observed in these analyses (all *P* > 0.05). (Table 2). The leave-one-out sensitivity analysis, as shown in Figures 5(d) and 6(d), demonstrated that the overall estimates were not disproportionately affected by any individual SNP. Additionally, the funnel plots in Figures 5(b) and 6(b) show no evidence of horizontal pleiotropy.

### 3.4. Genetic Prediction of RTIs for Risk of Plasma MPO Levels

To assess reverse causality, we extracted 13 and 7 SNPs independently linked to URTI and LRTI (ICU) in the primary MR study, respectively, with a significance of *P* < 5 × 10^–6^. Our supplementary MR investigation identified 11 and 7 SNPs substantially associated with URTI and LRTI (ICU) risk (*P* < 5 × 10^–6^). Information on genetic instruments is presented in Supplementary Tables S5–S7, and the detailed SNP filtering process is shown in Supplementary Tables S8 and S9. The F statistic of the instrument SNPs ranged from 3,997 to 21,953. The MR results indicated that there were no causal effects of URTI (beta = 0.021, 95% CI = −0.005–0.046, *P*=0.111; beta = −0.007, 95% CI = −0.119–0.106, *P*=0.905) or LRTI (ICU) (beta = 0.013, 95% CI = −0.001–0.026, *P*=0.068; beta = −0.006, 95% CI = −0.039–0.026, *P*=0.705) on plasma MPO levels using the IVW method with the random effect model (Figure 2(b)). There was no heterogeneity or pleiotropy in the sensitivity analysis of the reverse MR analyses. The results of the sensitivity analysis are shown in Table 2.

## 4. Discussion

Previous observational and clinical studies have shown an association between plasma MPO levels and RTIs, but the exact causal relationships have yet to be well established. In this study, we conducted a bidirectional MR analysis to systematically explore the causative relationships among plasma MPO levels and RTIs based on summary-level data from large-scale GWASs. We showed a direct causality between higher plasma MPO levels and a higher URTI and LRTI (ICU) risk for the first time. In contrast, the causal role of URTI and LRTI (ICU) on plasma MPO concentration was not supported in our MR analysis.

Observational studies have provided abundant evidence for the association between MPO and the risk of RTIs. For instance, in an analysis based on three pediatric patients with ARDS infected with H5N1 influenza and 31 non-H5N1 influenza-infected ARDS children, plasma MPO levels were higher in pediatric patients with ARDS infected with H5N1 influenza than in non-H5N1 influenza-infected ARDS children [13]. In addition, a prospective study of 279 individuals showed that plasma MPO levels in nonsevere (NS), severe (S), and postacute phase (PAP) COVID-19 patients were significantly different from the levels in healthy individuals, and plasma MPO levels had high diagnostic power for the disease severity of COVID-19 [26]. Moreover, Chang and Yao [12] conducted a prospective cohort study on adult acute exacerbation of chronic obstructive pulmonary disease (AECOPD) patients, suggesting that plasma MPO levels in patients with frequent AECOPD exacerbations were significantly higher than in patients with infrequent AECOPD exacerbations. Similar associations were reported for MPO levels in respiratory specimens and the risk of respiratory infection [27–30]. Our findings further highlight these associations. We found that plasma MPO concentration was genetically associated with increased risks of respiratory infections (URTI: OR = 1.135, 95% CI = 1.011–1.274, *P*=0.032; LRTI in the critical care units: OR = 1.323, 95% CI = 1.006–1.739, *P*=0.045). Unfortunately, null causal effects of URTI and LRTI (ICU) risk on plasma MPO concentration were observed in our MR study (URTI: beta = 0.021, 95% CI = −0.005–0.046, *P*=0.111; LRTI in the critical care units: beta = 0.013, 95% CI = −0.001–0.026, *P*=0.068).

Several possible pathophysiological mechanisms underlying the detrimental effect of plasma MPO levels on the risks of RTIs have been suggested by previous studies, including impaired neutrophil function, oxidative stress, and altered immune regulation [31–33]. MPO is an essential component of neutrophils, but excessive release of MPO can impair their function. Thus, elevated levels of MPO may lead to neutrophil dysfunction, making it more difficult for the immune system to clear respiratory pathogens effectively [31]. MPO and its oxidative products can directly harm the respiratory epithelium, increase airway epithelial permeability, compromise the integrity of the mucosal barrier, and decrease the mucociliary clearance mechanism, making it easier for pathogens to establish infections in the airways [32]. In addition, MPO has been shown to modulate the activity of various immune cells, such as macrophages and lymphocytes [33]. Dysregulation of immune responses due to elevated MPO levels can lead to an imbalance in the immune system, potentially impairing the body's ability to defend against respiratory pathogens effectively. Therefore, the detailed mechanism underlying the different associations of plasma MPO levels with RTIs and their subtypes warrants further study.

To the best of our knowledge, this study is the first to assess plasma MPO levels as a causal risk factor for RTIs using the MR design with data from a substantial number of individuals. There are several important public health significances and clinical implications. In the present MR study, we demonstrated the potential causal relationships between plasma MPO levels and incidence of URTI and LRTI (ICU) from the genetic insights, which might provide novel clues for preventing RTIs. According to our findings, plasma MPO levels could be a promising biomarker for identifying high-risk individuals for active surveillance and early intervention of RTIs. Furthermore, investigating whether targeting MPO could reduce the risk of RTIs is of clinical interest.

However, several limitations were also present. First, all GWAS data came from the European population. Whether our described findings would be consistent in other populations remains to be investigated. Second, the plasma MPO levels may be affected by both genes and the environment. However, our results can only explain the relationship between the changes in plasma MPO levels caused by genetic variation and infections [34]. Third, the GWAS database did not include detailed demographic characteristics or clinical data. Therefore, subgroup analysis cannot be further performed. Last, as mentioned in our previous method, three assumptions must be met to use variables as genetic instruments in MR analysis. If the first assumption is not satisfied, a “weak instrument problem,” such as weak statistical power and increased bias due to pleiotropic effects, occurs. The first assumption is tested by checking whether the *F*-statistic exceeds 10. Our study's *F*-statistic values were all ≥10, indicating no relevance assumption violation. However, due to the relatively small number of SNPs related to MPO we obtained, we did not validate Hypotheses 2 and 3, which may have led to some bias in our results.

## 5. Conclusion

Our two-sample MR study provides strong evidence for a causal relationship between plasma MPO levels and RTIs. We found that plasma MPO levels increased the risk of URTI and LRTI (ICU). However, there was no evidence of reverse causation. Our findings prompt future studies to investigate and confirm the role of MPO as a clinical biomarker that regulates the risk of RTIs and its potential role in therapeutic interventions.

## Figures and Tables

**Table 1:** Details of the GWASs included in the Mendelian randomization.

Event	GWAS ID	Consortium	Year	Population	Sample size
MPO	ebi-a-GCST90012031	Folkersen et al. [20] study	2020	European	21,758
MPO	prot-a-1930	INTERVAL study [21]	2018	European	3,301
URTI	ieu-b-5063	UK Biobank database	2021	European	2,795/483,689
LRTI (ICU)	ieu-b-4974	UK Biobank database	2021	European	585/430,780

MPO, myeloperoxidase; URTI, upper respiratory tract infection; LRTI (ICU), lower respiratory tract infection in the intensive care unit.

**Table 2:** Pleiotropy and heterogeneity test of the bidirectional Mendelian randomization study.

Exposure	Outcome	Pleiotropy test				Heterogeneity test					
		MR-Egger			PRESSO	MR-Egger			IVW		
		Intercept	SE	*P*-value	*P*-value	*Q*	*Q*_df	*Q*_*P* val	*Q*	*Q*_df	*Q*_*P* val
MPO^*∗*^	URTI	−0.005	0.012	0.674	0.972	14.526	26	0.965	14.707	27	0.973
MPO^*∗*^	LRTI (ICU)	−0.001	0.027	0.957	0.908	18.768	27	0.878	18.771	28	0.905
MPO#	URTI	−0.007	0.024	0.790	0.923	4.462	9	0.878	4.537	10	0.920
MPO#	LRTI (ICU)	−0.009	0.052	0.865	0.901	5.003	9	0.834	5.034	10	0.889
URTI	MPO^*∗*^	−0.008	0.009	0.406	0.972	3.719	11	0.977	4.466	12	0.973
LRTI (ICU)	MPO^*∗*^	0.007	0.021	0.749	0.945	1.570	5	0.905	1.684	6	0.946
URTI	MPO#	0.008	0.026	0.773	0.428	10.069	9	0.345	10.168	10	0.426
LRTI (ICU)	MPO#	−0.013	0.047	0.790	0.948	1.574	5	0.904	1.653	6	0.949

Horizontal pleiotropy analyses were conducted by MR-Egger regression and MR-PRESSO methods, and the results showed that there is no evidence of horizontal pleiotropy in IVs of infectious diseases (all *P* > 0.05). Heterogeneity tests were conducted by MR-Egger regression and IVW with the fixed model, and the results showed that there is no evidence of heterogeneity in IVs of infectious diseases (all *P* > 0.05). PRESSO, MR-Pleiotropy RESidual Sum and Outlier; IVW, inverse variance weighted method with the fixed effect model. MPO^*∗*^, MPO-related SNPs from the Folkersen et al. [20] study; MPO#, MPO-related SNPs from INTERVAL study [21].

## Data Availability

Summary information on the SNPs used as IVs in the primary MR study and the supplementary MR study are available in Supplementary Tables S1–S7. The characteristics of the SNP filtering process in the primary MR analysis and the supplementary MR study are available in Supplementary Tables S8 and S9. URTI and LRTI (ICU) data were obtained from the UK Biobank database. For the UK Biobank, applications for individual-level data can be made through the UK Biobank portal at https://www.ukbiobank.ac.uk/enable-yourresearch/apply-for-access.

## Abbreviations

MR: Mendelian randomization
IVW: Inverse variance weighted
GWAS: Genome-wide association study
SNPs: Single-nucleotide polymorphisms
IVs: Instrumental variables
MR-PRESSO: MR pleiotropy residual sum and outliers
OR: Odds ratio
CI: Confidence interval
MPO: Myeloperoxidase
RTIs: Respiratory tract infections
HOCl: Hypochlorous acid
ROS: Reactive oxygen species
URTI: Upper respiratory tract infection
LRTI (ICU): Lower respiratory tract infection in the intensive care unit
CES: Cardioembolic stroke
HF: Heart failure
AF: Atrial fibrillation
ARDS: Acute respiratory distress syndrome
AECOPD: Acute exacerbation of chronic obstructive pulmonary disease.

## Supplementary Materials

Supplementary Materials Supplementary Table S1: summary information of the SNPs used as IVs in the primary MR study between plasma MPO levels and URTI. Supplementary Table S2: summary information of the SNPs used as IVs in the supplementary MR study between plasma MPO levels and URTI. Supplementary Table S3: summary information of the SNPs used as IVs in the primary MR study between plasma MPO levels and LRTI (ICU). Supplementary Table S4: summary information of the SNPs used as IVs in the supplementary MR study between plasma MPO levels and LRTI (ICU). Supplementary Table S5: summary information of the SNPs used as IVs in the primary MR study between URTI and plasma MPO levels. Supplementary Table S6: summary information of the SNPs used as IVs in the supplementary MR study between URTI and plasma MPO levels. Supplementary Table S7: summary information of the SNPs used as IVs in the primary and supplementary MR studies between LRTI (ICU) and plasma MPO levels. Supplementary Table S8: characteristics of the SNPs filtering process in primary MR analysis. Supplementary Table S9: characteristics of the SNPs filtering process in supplementary MR analysis.

## References

[1] Tobler A., Miller C. W., Johnson K. R., Selsted M. E., Rovera G., Koeffler H. P. (1988). Regulation of gene expression of myeloperoxidase during myeloid differentiation. *Journal of Cellular Physiology*.

[2] Schultz J., Kaminker K. (1962). Myeloperoxidase of the leucocyte of normal human blood. I. Content and localization. *Archives of Biochemistry and Biophysics*.

[3] Bos A., Wever R., Roos D. (1978). Characterization and quantification of the peroxidase in human monocytes. *Biochimica et Biophysica Acta (BBA) - Enzymology*.

[4] Daugherty A., Dunn J. L., Rateri D. L., Heinecke J. W. (1994). Myeloperoxidase, a catalyst for lipoprotein oxidation, is expressed in human atherosclerotic lesions. *Journal of Clinical Investigation*.

[5] Nauseef W. M. (2007). How human neutrophils kill and degrade microbes: an integrated view. *Immunological Reviews*.

[6] Klebanoff S. J. (2005). Myeloperoxidase: friend and foe. *Journal of Leukocyte Biology*.

[7] GBD 2017 Disease and Injury Incidence and Prevalence Collaborators (2018). Global, regional, and national incidence, prevalence, and years lived with disability for 354 diseases and injuries for 195 countries and territories, 1990–2017: a systematic analysis for the global burden of disease study 2017. *The Lancet*.

[8] Davies M. J. (2021). Myeloperoxidase: mechanisms, reactions and inhibition as a therapeutic strategy in inflammatory diseases. *Pharmacology & Therapeutics*.

[9] Zhu L., Liu L., Zhang Y. (2018). High level of neutrophil extracellular traps correlates with poor prognosis of severe influenza A infection. *The Journal of Infectious Diseases*.

[10] Zuo Y., Yalavarthi S., Shi H. (2020). Neutrophil extracellular traps in COVID-19. *JCI Insight*.

[11] Fiorini G., Crespi S., Rinaldi M., Oberti E., Vigorelli R., Palmieri G. (2000). Serum ECP and MPO are increased during exacerbations of chronic bronchitis with airway obstruction. *Biomedicine & Pharmacotherapy*.

[12] Chang C., Yao W. (2014). Time course of inflammation resolution in patients with frequent exacerbations of chronic obstructive pulmonary disease. *Medical Science Monitor*.

[13] Phung T. T. B., Luong S. T., Kawachi S. (2011). Interleukin 12 and myeloperoxidase (MPO) in Vietnamese children with acute respiratory distress syndrome due to avian influenza (H5N1) infection. *Journal of Infection*.

[14] Bendib I., de Chaisemartin L., Granger V. (2019). Neutrophil extracellular traps are elevated in patients with pneumonia-related acute respiratory distress syndrome. *Anesthesiology*.

[15] Kothari N., Keshari R. S., Bogra J. (2011). Increased myeloperoxidase enzyme activity in plasma is an indicator of inflammation and onset of sepsis. *Journal of Critical Care*.

[16] Emdin C. A., Khera A. V., Kathiresan S. (2017). Mendelian randomization. *JAMA*.

[17] Zheng J., Baird D., Borges M.-C. (2017). Recent developments in Mendelian randomization studies. *Current Epidemiology Reports*.

[18] Wang Y., Jia Y., Xu Q. (2023). Association between myeloperoxidase and the risks of ischemic stroke, heart failure, and atrial fibrillation: a Mendelian randomization study. *Nutrition, Metabolism & Cardiovascular Diseases*.

[19] Skrivankova V. W., Richmond R. C., Woolf B. A. R. (2021). Strengthening the reporting of observational studies in epidemiology using Mendelian randomization: the STROBE-MR statement. *JAMA*.

[20] Folkersen L., Gustafsson S., Wang Q. (2020). Genomic and drug target evaluation of 90 cardiovascular proteins in 30,931 individuals. *Nature Metabolism*.

[21] Sun B. B., Maranville J. C., Peters J. E. (2018). Genomic atlas of the human plasma proteome. *Nature*.

[22] Zeng Y., Cao S., Yang H. (2023). Roles of gut microbiome in epilepsy risk: a Mendelian randomization study. *Frontiers in Microbiology*.

[23] Verbanck M., Chen C.-Y., Neale B., Do R. (2018). Detection of widespread horizontal pleiotropy in causal relationships inferred from Mendelian randomization between complex traits and diseases. *Nature Genetics*.

[24] Burgess S., Thompson S. G. (2017). Interpreting findings from Mendelian randomization using the MR-Egger method. *European Journal of Epidemiology*.

[25] Ong J.-S., MacGregor S. (2019). Implementing MR-PRESSO and GCTA-GSMR for pleiotropy assessment in Mendelian randomization studies from a practitioner’s perspective. *Genetic Epidemiology*.

[26] Pisareva E., Badiou S., Mihalovičová L. (2023). Persistence of neutrophil extracellular traps and anticardiolipin auto-antibodies in post-acute phase COVID-19 patients. *Journal of Medical Virology*.

[27] Jin J., Guo B., Zhang W. (2023). Diagnostic value of myeloperoxidase and eosinophil cationic protein in nasal secretions for endotypes of chronic rhinosinusitis. *European Archives of Oto-Rhino-Laryngology*.

[28] Cavallaro E. C., Liang K.-K., Lawrence M. D., Forsyth K. D., Dixon D.-L. (2017). Neutrophil infiltration and activation in bronchiolitic airways are independent of viral etiology. *Pediatric Pulmonology*.

[29] Chiu K. H.-Y., Yip C. C.-Y., Poon W.-S. (2023). Correlations of myeloperoxidase (MPO), adenosine deaminase (ADA), C–C motif chemokine 22 (CCL22), tumour necrosis factor alpha (TNF*α*) and interleukin-6 (IL-6) mRNA expression in the nasopharyngeal specimens with the diagnosis and severity of SARS-CoV-2 infections. *Emerging Microbes & Infections*.

[30] Bresser P., Out T. A., van Alphen L., Jansen H. M., Lutter R. (2000). Airway inflammation in nonobstructive and obstructive chronic bronchitis with chronic *Haemophilus influenzae* airway infection. *American Journal of Respiratory and Critical Care Medicine*.

[31] Rizo-Téllez S. A., Sekheri M., Filep J. G. (2022). Myeloperoxidase: regulation of neutrophil function and target for therapy. *Antioxidants*.

[32] Regelmann W. E., Schneider L. A., Fahrenkrug S. C. (1997). Proteinase-free myeloperoxidase increases airway epithelial permeability in a whole trachea model. *Pediatric Pulmonology*.

[33] Odobasic D., Kitching A. R., Holdsworth S. R. (2016). Neutrophil-mediated regulation of innate and adaptive immunity: the role of myeloperoxidase. *Journal of Immunology Research*.

[34] VanderWeele T. J., Tchetgen Tchetgen E. J., Cornelis M., Kraft P. (2014). Methodological challenges in Mendelian randomization. *Epidemiology*.

