# Supplementary material for: The Causal Relationship between Plasma Myeloperoxidase Levels and Respiratory Tract Infections: A Bidirectional Mendelian Randomization Study
Source: Mediators Inflamm. 2024 Mar 28;2024:6626706. doi: 10.1155/2024/6626706 (PMC10994701; doi:10.1155/2024/6626706)
Supplement: Supplementary Materials — Supplementary Table S1: summary information of the SNPs used as IVs in the primary MR study between plasma MPO levels and URTI. Supplementary Table S2: summary information of the SNPs used as IVs in the supplementary MR study between plasma MPO levels and URTI. Supplementary Table S3: summary information of the SNPs used as IVs in the primary MR study between plasma MPO levels and LRTI (ICU). Supplementary Table S4: summary information of the SNPs used as IVs in the supplementary MR study between plasma MPO levels and LRTI (ICU). Supplementary Table S5: summary information of the SNPs used as IVs in the primary MR study between URTI and plasma MPO levels. Supplementary Table S6: summary information of the SNPs used as IVs in the supplementary MR study between URTI and plasma MPO levels. Supplementary Table S7: summary information of the SNPs used as IVs in the primary and supplementary MR studies between LRTI (ICU) and plasma MPO levels. Supplementary Table S8: characteristics of the SNPs filtering process in primary MR analysis. Supplementary Table S9: characteristics of the SNPs filtering process in supplementary MR analysis. [file 6626706.f1.doc]

**Supplementary Material**

**Supplementary Table S1:** Summary information of the SNPs used as IVs in the primary MR study between plasma MPO levels and URTI.

**Supplementary Table S2:** Summary information of the SNPs used as IVs in the supplementary MR study between plasma MPO levels and URTI.

**Supplementary Table S3:** Summary information of the SNPs used as IVs in the primary MR study between plasma MPO levels and LRTI (ICU).

**Supplementary Table S4:** Summary information of the SNPs used as IVs in the supplementary MR study between plasma MPO levels and LRTI (ICU).

**Supplementary Table S5:** Summary information of the SNPs used as IVs in the primary MR study between URTI and plasma MPO levels.

**Supplementary Table S6:** Summary information of the SNPs used as IVs in the supplementary MR study between URTI and plasma MPO levels.

**Supplementary Table S7:** Summary information of the SNPs used as IVs in the primary and supplementary MR studies between LRTI (ICU) and plasma MPO levels.

**Supplementary Table S8:** Characteristics of the SNPs filtering process in primary MR analysis.

**Supplementary Table S9:** Characteristics of the SNPs filtering process in supplementary MR analysis.

**Supplementary Table S1:** Summary information of the SNPs used as IVs in the primary MR study between plasma MPO levels and URTI.

| SNP | chr | pos | effect_allele | other_allele | eaf.exposure | beta.exposure | se.exposure | pval.exposure | beta.outcome | se.outcome | pval.outcome | samplesize | F |
| --- | --- | --- | --- | --- | --- | --- | --- | --- | --- | --- | --- | --- | --- |
| rs10103048 | 8 | 130602281 | C | A | 0.585 | -0.074 | 0.010 | 1.14E-14 | 0.027 | 0.029 | 0.340 | 21758 | 58 |
| rs10282219 | 7 | 123612325 | G | A | 0.289 | -0.049 | 0.011 | 2.95E-06 | 0.024 | 0.030 | 0.422 | 21758 | 22 |
| rs10418923 | 19 | 16248311 | G | A | 0.708 | 0.065 | 0.011 | 7.50E-10 | 0.015 | 0.031 | 0.626 | 21758 | 38 |
| rs10753459 | 1 | 229575764 | A | G | 0.175 | 0.109 | 0.012 | 1.43E-19 | 0.052 | 0.037 | 0.160 | 21758 | 75 |
| rs11199938 | 10 | 123135645 | C | T | 0.347 | -0.058 | 0.011 | 2.20E-07 | 0.004 | 0.030 | 0.883 | 21758 | 33 |
| rs1150754 | 6 | 32050758 | T | C | 0.152 | -0.075 | 0.013 | 5.87E-09 | -0.010 | 0.037 | 0.792 | 21758 | 31 |
| rs117148585 | 8 | 79498844 | A | T | 0.035 | 0.148 | 0.030 | 6.79E-07 | 0.105 | 0.081 | 0.195 | 21758 | 32 |
| rs12589195 | 14 | 93614062 | A | C | 0.245 | -0.057 | 0.011 | 1.65E-07 | 0.007 | 0.033 | 0.843 | 21758 | 26 |
| rs13032049 | 2 | 63581507 | G | A | 0.278 | -0.050 | 0.011 | 2.93E-06 | -0.006 | 0.031 | 0.859 | 21758 | 21 |
| rs13107325 | 4 | 103188709 | T | C | 0.066 | 0.108 | 0.022 | 7.10E-07 | 0.042 | 0.053 | 0.426 | 21758 | 31 |
| rs138285555 | 10 | 94262194 | G | C | 0.030 | 0.165 | 0.035 | 2.03E-06 | 0.058 | 0.086 | 0.499 | 21758 | 34 |
| rs150918492 | 3 | 128389659 | A | G | 0.263 | -0.072 | 0.012 | 5.81E-09 | -0.019 | 0.032 | 0.543 | 21758 | 44 |
| rs2167252 | 8 | 56771296 | G | C | 0.783 | -0.053 | 0.011 | 2.36E-06 | 0.013 | 0.034 | 0.707 | 21758 | 21 |
| rs25913 | 5 | 108993934 | C | T | 0.337 | 0.049 | 0.011 | 3.23E-06 | -0.019 | 0.030 | 0.530 | 21758 | 24 |
| rs314256 | 17 | 7089652 | G | C | 0.364 | 0.046 | 0.010 | 4.23E-06 | -0.024 | 0.029 | 0.404 | 21758 | 22 |
| rs34097845 | 17 | 56358429 | T | C | 0.057 | -0.349 | 0.019 | 2.70E-73 | -0.093 | 0.062 | 0.136 | 21758 | 287 |
| rs417463 | 21 | 19155218 | A | G | 0.470 | -0.049 | 0.010 | 3.23E-07 | -0.004 | 0.028 | 0.883 | 21758 | 26 |
| rs4942786 | 13 | 49389309 | G | A | 0.516 | 0.048 | 0.011 | 4.20E-06 | 0.009 | 0.028 | 0.758 | 21758 | 25 |
| rs564124209 | 15 | 60473685 | A | C | 0.017 | -0.334 | 0.063 | 1.01E-07 | 0.000 | 0.127 | 0.999 | 21758 | 82 |
| rs56871666 | 18 | 50226797 | G | A | 0.103 | 0.070 | 0.015 | 2.53E-06 | 0.031 | 0.045 | 0.501 | 21758 | 20 |
| rs6034875 | 20 | 17627727 | A | G | 0.582 | -0.075 | 0.010 | 4.00E-15 | -0.034 | 0.028 | 0.234 | 21758 | 60 |
| rs62442278 | 7 | 6874714 | T | A | 0.187 | 0.073 | 0.014 | 1.81E-07 | -0.004 | 0.037 | 0.915 | 21758 | 36 |
| rs66493857 | 17 | 4178196 | G | A | 0.672 | -0.054 | 0.012 | 4.61E-06 | -0.028 | 0.030 | 0.344 | 21758 | 28 |
| rs74343467 | 2 | 43772121 | T | C | 0.544 | -0.054 | 0.011 | 3.97E-07 | -0.019 | 0.028 | 0.515 | 21758 | 31 |
| rs7502971 | 17 | 38186501 | A | C | 0.370 | -0.056 | 0.010 | 9.22E-09 | -0.045 | 0.029 | 0.116 | 21758 | 32 |
| rs757081 | 11 | 17351683 | G | C | 0.336 | 0.098 | 0.010 | 4.26E-23 | -0.019 | 0.030 | 0.524 | 21758 | 94 |
| rs78978525 | 1 | 219353084 | G | A | 0.042 | -0.148 | 0.029 | 4.21E-07 | 0.003 | 0.068 | 0.961 | 21758 | 38 |
| rs8178414 | 17 | 56345363 | T | C | 0.022 | -0.436 | 0.039 | 4.86E-29 | 0.086 | 0.124 | 0.485 | 21758 | 178 |

Chr, chromosome; pos, position; eaf, eaf, effect allele frequency; se, Standard error.

**Supplementary Table S2:** Summary information of the SNPs used as IVs in the supplementary MR study between plasma MPO levels and URTI.

| SNP | chr | pos | effect_allele | other_allele | eaf.exposure | beta.exposure | se.exposure | pval.exposure | beta.outcome | se.outcome | pval.outcome | samplesize | F |
| --- | --- | --- | --- | --- | --- | --- | --- | --- | --- | --- | --- | --- | --- |
| rs11980487 | 7 | 6545619 | C | T | 0.701 | 0.135 | 0.027 | 6.76E-07 | 0.002 | 0.031 | 0.935 | 3301 | 25 |
| rs12467999 | 2 | 12159408 | A | G | 0.213 | -0.138 | 0.030 | 4.07E-06 | -0.005 | 0.034 | 0.877 | 3301 | 21 |
| rs13036464 | 20 | 17669186 | C | G | 0.400 | 0.140 | 0.025 | 2.69E-08 | 0.020 | 0.028 | 0.471 | 3301 | 31 |
| rs138531759 | 8 | 14596257 | C | T | 0.016 | -0.475 | 0.103 | 3.80E-06 | -0.084 | 0.109 | 0.441 | 3301 | 24 |
| rs17027306 | 2 | 41439781 | C | G | 0.040 | 0.331 | 0.069 | 1.91E-06 | -0.008 | 0.080 | 0.925 | 3301 | 28 |
| rs180698348 | 7 | 36506512 | A | T | 0.107 | 0.185 | 0.040 | 4.37E-06 | 0.045 | 0.048 | 0.347 | 3301 | 22 |
| rs34097845 | 17 | 56358429 | T | C | 0.067 | -0.574 | 0.050 | 8.51E-31 | -0.093 | 0.062 | 0.136 | 3301 | 142 |
| rs4694141 | 4 | 73701313 | C | T | 0.366 | 0.121 | 0.026 | 3.31E-06 | 0.044 | 0.028 | 0.122 | 3301 | 22 |
| rs4925496 | 1 | 229633371 | G | A | 0.264 | 0.188 | 0.028 | 1.70E-11 | 0.051 | 0.032 | 0.111 | 3301 | 46 |
| rs556811945 | 1 | 26974072 | A | G | 0.024 | -0.462 | 0.095 | 9.77E-07 | -0.131 | 0.115 | 0.255 | 3301 | 33 |
| rs56213534 | 11 | 17350394 | G | A | 0.305 | 0.154 | 0.027 | 1.26E-08 | -0.017 | 0.030 | 0.581 | 3301 | 33 |

**Supplementary Table S3:** Summary information of the SNPs used as IVs in the primary MR study between plasma MPO levels and LRTI (ICU).

| SNP | chr | pos | effect_allele | other_allele | eaf.exposure | beta.exposure | se.exposure | pval.exposure | beta.outcome | se.outcome | pval.outcome | samplesize | F |
| --- | --- | --- | --- | --- | --- | --- | --- | --- | --- | --- | --- | --- | --- |
| rs10103048 | 8 | 130602281 | C | A | 0.585 | -0.074 | 0.010 | 1.14E-14 | 0.031 | 0.062 | 0.616 | 21758 | 58 |
| rs10282219 | 7 | 123612325 | G | A | 0.289 | -0.049 | 0.011 | 2.95E-06 | -0.109 | 0.066 | 0.096 | 21758 | 22 |
| rs10753459 | 1 | 229575764 | A | G | 0.175 | 0.109 | 0.012 | 1.43E-19 | 0.048 | 0.080 | 0.543 | 21758 | 75 |
| rs11199938 | 10 | 123135645 | C | T | 0.347 | -0.058 | 0.011 | 2.20E-07 | -0.005 | 0.064 | 0.933 | 21758 | 33 |
| rs1150754 | 6 | 32050758 | T | C | 0.152 | -0.075 | 0.013 | 5.87E-09 | 0.071 | 0.080 | 0.379 | 21758 | 31 |
| rs117148585 | 8 | 79498844 | A | T | 0.035 | 0.148 | 0.030 | 6.79E-07 | 0.101 | 0.173 | 0.560 | 21758 | 32 |
| rs12589195 | 14 | 93614062 | A | C | 0.245 | -0.057 | 0.011 | 1.65E-07 | 0.004 | 0.071 | 0.958 | 21758 | 26 |
| rs13032049 | 2 | 63581507 | G | A | 0.278 | -0.050 | 0.011 | 2.93E-06 | -0.027 | 0.067 | 0.692 | 21758 | 21 |
| rs13107325 | 4 | 103188709 | T | C | 0.066 | 0.108 | 0.022 | 7.10E-07 | -0.103 | 0.115 | 0.372 | 21758 | 31 |
| rs138285555 | 10 | 94262194 | G | C | 0.030 | 0.165 | 0.035 | 2.03E-06 | 0.177 | 0.187 | 0.344 | 21758 | 34 |
| rs145096717 | 13 | 28761592 | A | G | 0.016 | 0.279 | 0.059 | 2.52E-06 | -0.223 | 0.290 | 0.443 | 21758 | 53 |
| rs150918492 | 3 | 128389659 | A | G | 0.263 | -0.072 | 0.012 | 5.81E-09 | -0.002 | 0.068 | 0.981 | 21758 | 44 |
| rs188737126 | 15 | 24518476 | G | A | 0.031 | 0.202 | 0.041 | 7.50E-07 | 0.010 | 0.262 | 0.969 | 21758 | 54 |
| rs2167252 | 8 | 56771296 | G | C | 0.783 | -0.053 | 0.011 | 2.36E-06 | -0.026 | 0.073 | 0.725 | 21758 | 21 |
| rs25913 | 5 | 108993934 | C | T | 0.337 | 0.049 | 0.011 | 3.23E-06 | -0.008 | 0.065 | 0.908 | 21758 | 24 |
| rs314256 | 17 | 7089652 | G | C | 0.364 | 0.046 | 0.010 | 4.23E-06 | 0.028 | 0.063 | 0.656 | 21758 | 22 |
| rs34097845 | 17 | 56358429 | T | C | 0.057 | -0.349 | 0.019 | 2.70E-73 | -0.168 | 0.134 | 0.207 | 21758 | 287 |
| rs34723959 | 7 | 88858393 | A | G | 0.022 | 0.209 | 0.042 | 7.03E-07 | 0.188 | 0.211 | 0.372 | 21758 | 41 |
| rs417463 | 21 | 19155218 | A | G | 0.470 | -0.049 | 0.010 | 3.23E-07 | 0.041 | 0.060 | 0.492 | 21758 | 26 |
| rs4942786 | 13 | 49389309 | G | A | 0.516 | 0.048 | 0.011 | 4.20E-06 | 0.082 | 0.061 | 0.175 | 21758 | 25 |
| rs564124209 | 15 | 60473685 | A | C | 0.017 | -0.334 | 0.063 | 1.01E-07 | -0.483 | 0.278 | 0.082 | 21758 | 82 |
| rs56871666 | 18 | 50226797 | G | A | 0.103 | 0.070 | 0.015 | 2.53E-06 | -0.012 | 0.098 | 0.903 | 21758 | 20 |
| rs6034875 | 20 | 17627727 | A | G | 0.582 | -0.075 | 0.010 | 4.00E-15 | -0.072 | 0.061 | 0.239 | 21758 | 60 |
| rs62442278 | 7 | 6874714 | T | A | 0.187 | 0.073 | 0.014 | 1.81E-07 | -0.051 | 0.080 | 0.525 | 21758 | 36 |
| rs66493857 | 17 | 4178196 | G | A | 0.672 | -0.054 | 0.012 | 4.61E-06 | -0.007 | 0.064 | 0.909 | 21758 | 28 |
| rs7502971 | 17 | 38186501 | A | C | 0.370 | -0.056 | 0.010 | 9.22E-09 | -0.093 | 0.062 | 0.135 | 21758 | 32 |
| rs757081 | 11 | 17351683 | G | C | 0.336 | 0.098 | 0.010 | 4.26E-23 | 0.011 | 0.064 | 0.868 | 21758 | 94 |
| rs78978525 | 1 | 219353084 | G | A | 0.042 | -0.148 | 0.029 | 4.21E-07 | 0.220 | 0.147 | 0.134 | 21758 | 38 |
| rs8178414 | 17 | 56345363 | T | C | 0.022 | -0.436 | 0.039 | 4.86E-29 | -0.094 | 0.268 | 0.726 | 21758 | 178 |

**Supplementary Table S4:** Summary information of the SNPs used as IVs in the supplementary MR study between plasma MPO levels and LRTI (ICU).

| SNP | chr | pos | effect_allele | other_allele | eaf.exposure | beta.exposure | se.exposure | pval.exposure | beta.outcome | se.outcome | pval.outcome | samplesize | F |
| --- | --- | --- | --- | --- | --- | --- | --- | --- | --- | --- | --- | --- | --- |
| rs11980487 | 7 | 6545619 | C | T | 0.701 | 0.135 | 0.027 | 6.76E-07 | -0.001 | 0.066 | 0.994 | 3301 | 25 |
| rs12467999 | 2 | 12159408 | A | G | 0.213 | -0.138 | 0.030 | 4.07E-06 | 0.013 | 0.073 | 0.856 | 3301 | 21 |
| rs13036464 | 20 | 17669186 | C | G | 0.400 | 0.140 | 0.025 | 2.69E-08 | 0.075 | 0.061 | 0.219 | 3301 | 31 |
| rs138531759 | 8 | 14596257 | C | T | 0.016 | -0.475 | 0.103 | 3.80E-06 | -0.302 | 0.234 | 0.196 | 3301 | 24 |
| rs17027306 | 2 | 41439781 | C | G | 0.040 | 0.331 | 0.069 | 1.91E-06 | -0.147 | 0.176 | 0.403 | 3301 | 28 |
| rs180698348 | 7 | 36458285 | A | T | 0.107 | 0.185 | 0.040 | 4.37E-06 | 0.000 | 0.101 | 1.000 | 3301 | 22 |
| rs34097845 | 17 | 56358429 | T | C | 0.067 | -0.574 | 0.050 | 8.51E-31 | -0.168 | 0.134 | 0.207 | 3301 | 142 |
| rs4694141 | 4 | 73701313 | C | T | 0.366 | 0.121 | 0.026 | 3.31E-06 | 0.030 | 0.062 | 0.620 | 3301 | 22 |
| rs4925496 | 1 | 229633371 | G | A | 0.264 | 0.188 | 0.028 | 1.70E-11 | 0.083 | 0.069 | 0.232 | 3301 | 46 |
| rs556811945 | 1 | 26974072 | A | G | 0.024 | -0.462 | 0.095 | 9.77E-07 | 0.123 | 0.251 | 0.624 | 3301 | 33 |
| rs56213534 | 11 | 17350394 | G | A | 0.305 | 0.154 | 0.027 | 1.26E-08 | -0.001 | 0.066 | 0.988 | 3301 | 33 |

**Supplementary Table S5:** Summary information of the SNPs used as IVs in the primary MR study between URTI and plasma MPO levels.

| SNP | chr | pos | effect_allele | other_allele | eaf.exposure | beta.exposure | se.exposure | pval.exposure | beta.outcome | se.outcome | pval.outcome | samplesize | F |
| --- | --- | --- | --- | --- | --- | --- | --- | --- | --- | --- | --- | --- | --- |
| rs10832984 | 11 | 2991668 | T | A | 0.585 | -0.157 | 0.029 | 4.29E-08 | -0.157 | 0.010 | 0.378 | 486484 | 5858 |
| rs12743974 | 1 | 67708357 | A | G | 0.415 | -0.130 | 0.028 | 4.91E-06 | -0.130 | 0.010 | 0.407 | 486484 | 3997 |
| rs147403669 | 5 | 85400540 | A | G | 0.010 | -0.685 | 0.141 | 1.19E-06 | -0.685 | 0.057 | 0.284 | 486484 | 4553 |
| rs2032156 | 21 | 20641655 | G | A | 0.315 | 0.143 | 0.030 | 2.04E-06 | 0.143 | 0.011 | 0.513 | 486484 | 4353 |
| rs2450153 | 8 | 52971672 | G | A | 0.229 | -0.153 | 0.033 | 4.55E-06 | -0.153 | 0.012 | 0.638 | 486484 | 4051 |
| rs2480049 | 1 | 14788056 | G | C | 0.398 | -0.131 | 0.029 | 4.93E-06 | -0.131 | 0.011 | 0.789 | 486484 | 4048 |
| rs28853654 | 7 | 55424356 | A | G | 0.437 | -0.133 | 0.028 | 2.68E-06 | -0.133 | 0.011 | 0.438 | 486484 | 4274 |
| rs6139675 | 20 | 5235541 | T | C | 0.071 | -0.313 | 0.065 | 1.31E-06 | -0.313 | 0.025 | 0.983 | 486484 | 6406 |
| rs62060390 | 17 | 55465262 | T | C | 0.050 | 0.302 | 0.064 | 2.31E-06 | 0.302 | 0.024 | 0.817 | 486484 | 4261 |
| rs76017926 | 2 | 32165672 | C | A | 0.015 | 0.547 | 0.114 | 1.66E-06 | 0.547 | 0.051 | 0.407 | 486484 | 4458 |
| rs785288 | 2 | 53016153 | A | G | 0.943 | -0.296 | 0.061 | 1.22E-06 | -0.296 | 0.018 | 0.497 | 486484 | 4649 |
| rs79904875 | 21 | 43080950 | T | C | 0.038 | 0.348 | 0.073 | 2.05E-06 | 0.348 | 0.030 | 0.744 | 486484 | 4361 |
| rs957140 | 11 | 89201627 | A | G | 0.445 | -0.136 | 0.028 | 1.12E-06 | -0.136 | 0.010 | 0.702 | 486484 | 4499 |

**Supplementary Table S6:** Summary information of the SNPs used as IVs in the supplementary MR study between URTI and plasma MPO levels.

| SNP | chr | pos | effect_allele | other_allele | eaf.exposure | beta.exposure | se.exposure | pval.exposure | beta.outcome | se.outcome | pval.outcome | samplesize | F |
| --- | --- | --- | --- | --- | --- | --- | --- | --- | --- | --- | --- | --- | --- |
| rs10832984 | 11 | 2991668 | T | A | 0.585 | -0.157 | 0.029 | 4.29E-08 | 0.004 | 0.026 | 0.871 | 486484 | 5858 |
| rs12743974 | 1 | 67708357 | A | G | 0.415 | -0.130 | 0.028 | 4.91E-06 | -0.024 | 0.025 | 0.355 | 486484 | 3997 |
| rs147403669 | 5 | 85400540 | A | G | 0.010 | -0.685 | 0.141 | 1.19E-06 | -0.128 | 0.141 | 0.363 | 486484 | 4553 |
| rs2032156 | 21 | 20641655 | G | A | 0.315 | 0.143 | 0.030 | 2.04E-06 | -0.012 | 0.027 | 0.646 | 486484 | 4353 |
| rs2450153 | 8 | 52971672 | G | A | 0.229 | -0.153 | 0.033 | 4.55E-06 | -0.017 | 0.030 | 0.575 | 486484 | 4051 |
| rs2480049 | 1 | 14788056 | G | C | 0.398 | -0.131 | 0.029 | 4.93E-06 | 0.008 | 0.026 | 0.776 | 486484 | 4048 |
| rs28853654 | 7 | 55424356 | A | G | 0.437 | -0.133 | 0.028 | 2.68E-06 | -0.045 | 0.025 | 0.076 | 486484 | 4274 |
| rs62060390 | 17 | 55465262 | T | C | 0.050 | 0.302 | 0.064 | 2.31E-06 | -0.005 | 0.058 | 0.933 | 486484 | 4261 |
| rs76017926 | 2 | 32165672 | C | A | 0.015 | 0.547 | 0.114 | 1.66E-06 | -0.067 | 0.101 | 0.501 | 486484 | 4458 |
| rs79904875 | 21 | 43080950 | T | C | 0.038 | 0.348 | 0.073 | 2.05E-06 | -0.053 | 0.067 | 0.427 | 486484 | 4361 |
| rs957140 | 11 | 89201627 | A | G | 0.445 | -0.136 | 0.028 | 1.12E-06 | 0.047 | 0.025 | 0.056 | 486484 | 4499 |

**Supplementary Table S7:** Summary information of the SNPs used as IVs in the primary and supplementary MR studies between LRTI (ICU) and plasma MPO levels.

| SNP | chr | pos | effect_allele | other_allele | eaf.exposure | beta.exposure | se.exposure | pval.exposure | beta.outcome | se.outcome | pval.outcome | samplesize | F |
| --- | --- | --- | --- | --- | --- | --- | --- | --- | --- | --- | --- | --- | --- |
| rs114615031 | 4 | 2541879 | T | C | 0.024 | 0.898 | 0.196 | 4.79E-06 | 0.032 | 0.049 | 0.514 | 431365 | 17293 |
| rs12254725 | 10 | 61861235 | T | C | 0.068 | -0.577 | 0.121 | 1.93E-06 | -0.021 | 0.017 | 0.218 | 431365 | 18883 |
| rs34473406 | 7 | 75500398 | G | T | 0.080 | 0.574 | 0.115 | 5.76E-07 | -0.002 | 0.018 | 0.917 | 431365 | 21953 |
| rs35640922 | 12 | 131041063 | G | A | 0.120 | -0.450 | 0.093 | 1.39E-06 | 0.000 | 0.015 | 0.990 | 431365 | 19283 |
| rs56175249 | 9 | 15069389 | A | G | 0.143 | 0.414 | 0.088 | 2.81E-06 | 0.010 | 0.015 | 0.483 | 431365 | 18826 |
| rs632338 | 13 | 98961581 | T | C | 0.815 | 0.376 | 0.078 | 1.50E-06 | 0.006 | 0.015 | 0.716 | 431365 | 19241 |
| rs73021304 | 4 | 190467364 | C | G | 0.009 | 1.524 | 0.326 | 2.93E-06 | -0.010 | 0.050 | 0.845 | 431365 | 18441 |

**Supplementary Table S8:** Characteristics of the SNPs filtering process in primary MR analysis.

| Exposure | Outcome | SNPs correlated with exposure (*P* < 5 × 10^−6^) | SNPs not present in the outcome GWAS | Proxy SNPs (for SNPs not in outcome) | Palindromic and  incompatible SNPs | Horizontal pleiotropy | F ≤ 10 | nSNPs in MR analysis |
| --- | --- | --- | --- | --- | --- | --- | --- | --- |
| MPO | URTI | 33 | rs28693443  rs564124209 | rs28565024 (rs564124209) | rs4766578 | rs34723959  rs145096717  rs188737126 | NA | 28 |
| MPO | LRTI(ICU) | 33 | rs28693443  rs564124209 | rs28565024 (rs564124209) | rs4766578 | rs74343467  rs10418923 | NA | 29 |
| URTI | MPO | 13 | NA | NA | NA | NA | NA | 13 |
| LRTI(ICU) | MPO | 7 | NA | NA | NA | NA | NA | 7 |

**Supplementary Table S9:** Characteristics of the SNPs filtering process in supplementary MR analysis.

| Exposure | Outcome | SNPs correlated with exposure (*P* < 5 × 10^−6^) | SNPs not present in the outcome GWAS data | Proxy SNPs (for SNPs not in outcome) | Palindromic and  incompatible SNPs | Horizontal pleiotropy | F ≤ 10 | nSNPs in MR analysis |
| --- | --- | --- | --- | --- | --- | --- | --- | --- |
| MPO | URTI | 13 | rs145360019  rs13036464  rs180698348  rs4694141  rs56213534 | rs6044953 (rs13036464)  rs17291859 (rs180698348)  rs6845164 (rs4694141)  rs1073443 (rs56213534) | rs1991866 | NA | NA | 11 |
| MPO | LRTI(ICU) | 13 | rs145360019  rs13036464  rs180698348  rs4694141  rs56213534 | rs6044953 (rs13036464)  rs17291859 (rs180698348)  rs6845164 (rs4694141)  rs1073443 (rs56213534) | rs1991866 | NA | NA | 11 |
| URTI | MPO | 13 | rs6139675 | NA | NA | rs785288 | NA | 11 |
| LRTI(ICU) | MPO | 7 | NA | NA | NA | NA | NA | 7 |
